# Editorial: Critical complications in pediatric oncology and hematopoietic cell transplant – how far we have come and how much further we must go

**DOI:** 10.3389/fonc.2023.1148321

**Published:** 2023-02-22

**Authors:** Asya Agulnik, Kris M. Mahadeo, Marie E. Steiner, Jennifer Ann McArthur

**Affiliations:** ^1^ Department of Global Pediatric Medicine, St. Jude Children’s Research Hospital, Memphis, TN, United States; ^2^ Division of Critical Care, Department of Pediatrics, St. Jude Children's Research Hospital, Memphis, TN, United States; ^3^ Division of Pediatric Transplantation and Cellular Therapy, Duke University School of Medicine, Durham, NC, United States; ^4^ Division of Pediatric Hematology Oncology, M Health Fairview Masonic Children’s Hospital, Minneapolis, MN, United States

**Keywords:** pediatric cancer, pediatric critical care, hematopoietic cell transplant, pediatric oncology and hematology, CAR (chimeric antigen receptor) T cells

## History of pediatric onco-critical care

The evolving experience and expertise in caring for critically ill pediatric oncology, hematopoietic cell transplant (HCT), and cellular therapies (CT) patients is a very recent effort. This topic, however, carries behind it tremendous energy and enthusiasm as evidenced by the numerous quality submissions to this Research Topic, “Critical Complications in Pediatric Oncology and Hematopoietic Cell Transplantation”.

Pediatric HCT was first successfully performed by the Robert A. Good team in 1968 to treat a non-malignant disorder (X-linked severe combined immune deficiency) (1) followed by expanded indications to bone marrow malignancies and failure (2–4). In this era, pediatric critical care was also early in its infancy but was available to patients with sepsis/septic shock, hemorrhage, and respiratory insufficiency/failure. Pediatric renal replacement therapies were limited, tunneled central venous access was in development, and non-invasive positive pressure was still decades away. Initial hematopoietic growth factors, anti-viral agents, and infection prophylaxis were undergoing study in clinical trials.

Mortality in this early era in both adult and pediatric oncology and HCT patients with septic shock and/or acute respiratory failure was dismal, with mortality rates exceeding 80% (5–9). Indeed, one published series from this early period stated: “Intensive respiratory care is effective for patients with readily reversible causes of respiratory failure, but is generally futile for patients with progressive interstitial pneumonia” and “We also recommend providing bone-marrow transplant patients with realistic prognostic estimates … before transfer to the intensive care unit … This approach may reduce the amount of futile care” (10).

Patients transplanted in the 1980s and 1990s experienced more variable mortality and in the next 20 years, patient overall mortality improved to 40 to 60%. In the 21^st^ century, mortality has further improved in certain patient subsets, but remains substantial for those requiring mechanical ventilation, with severe pulmonary pathology as a cause for intubation, and with multi-organ failure (11, 12). However, most recently, even extracorporeal membrane oxygenation (ECMO) support for HCT patients is being considered and successfully implemented (13–15). Clearly, collaboration between oncology, HCT, and Pediatric Intensive Care Unit (PICU) clinicians is continuing to impact patient outcomes. Considerable work, however, remains to be done, as evidenced by the variety and volume of manuscripts submitted to this Research Topic.

## Current focus of pediatric onco-critical care—this research topic

**Figure 1:**
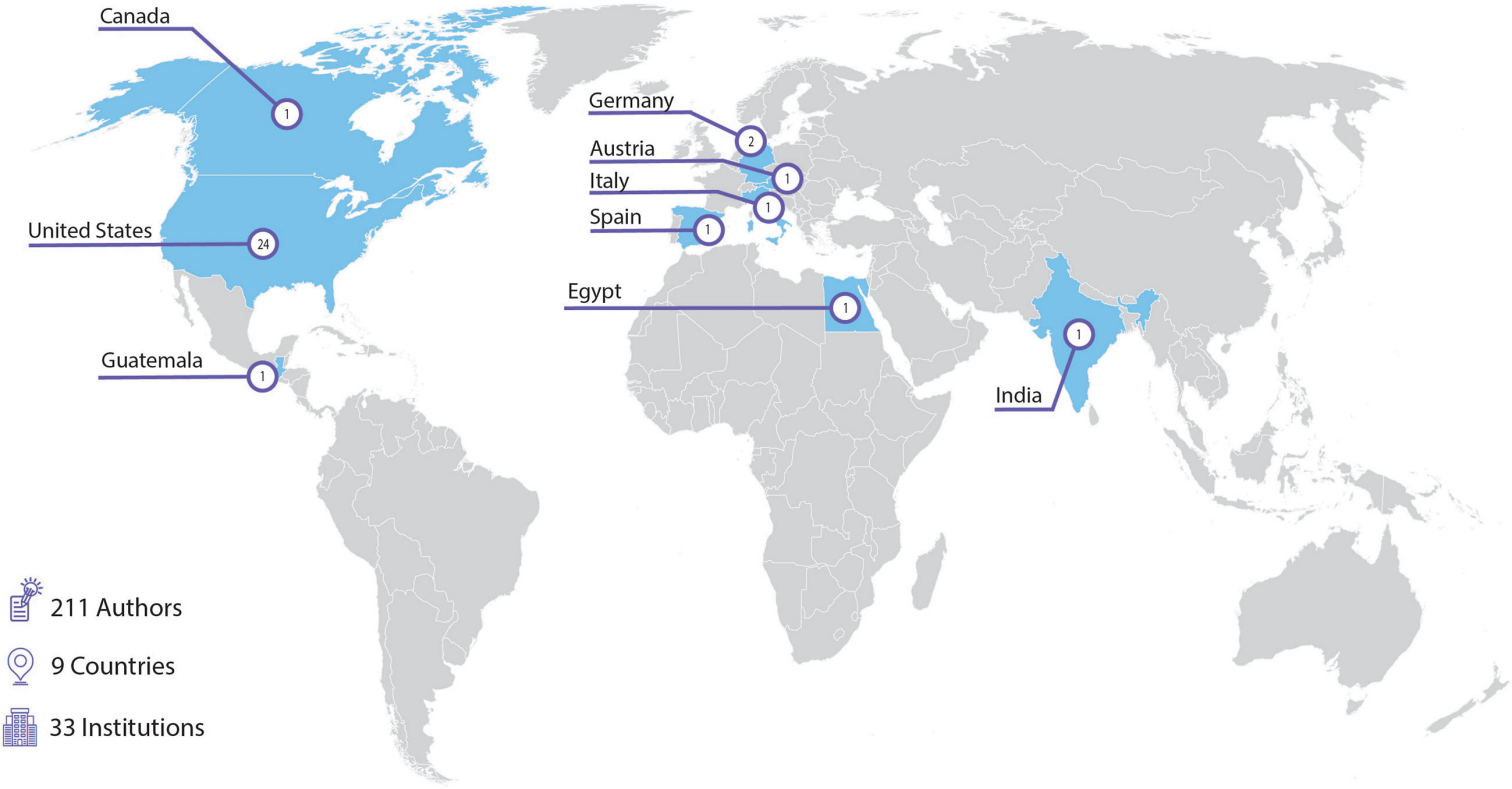
Institutions contributing accepted articles to the “Critical Complications in Pediatric Oncology and Hematopoietic Cell Transplantation” research topic (30 articles).

Articles published in this Research Topic demonstrates how far the field of Pediatric Onco-Critical Care has moved over the past 50 years. This Research Topic received a robust response in paper submissions, resulting in 30 articles by 211 authors from 33 institutions in 9 countries worldwide (**Figure 1**). As of January 2023, the Research Topic website has received over 90,000 views and over 21,000 downloads. Enthusiasm for this work continues; we have recently launched a second volume for this Research Topic, with 6 new articles published to date.

### Improving patient outcomes

In this collection, Pechlaner et al. performed a retrospective study of outcomes in pediatric hematology/oncology patients admitted to their ICU between 2009-2019. They showed PICU mortality to be 11% overall, though it was higher for those that required invasive mechanical ventilation (IMV), ECMO, and IMV with continuous renal replacement therapy (CRRT) at 34.5%, 42.9% and 53.8% respectively. Additionally, researchers found a significant increase in PICU admissions over the years after the implementation of a bedside pediatric early warning system (PEWS). The authors hypothesized that the institution of PEWS enabled their teams to recognize critical illness earlier and allow for earlier and more frequent transfer to the PICU. Mortality rates described in this study are similar to outcomes recently published from the Virtual PICU Systems (VPS) database (16) and add credence to the argument that outcomes are improving in this high-risk population.

### Novel therapies and interventions

Over the last decades, the field of pediatric hematology-oncology has advanced extensively to offer novel therapeutics for childhood cancer, moving childhood cancer from a universally fatal disease to a survival of greater than 80% in high-resource settings (17). These therapies, however, carry unique risks of toxicity, and optimum survival for patients can only be achieved with delivery of excellent supportive care. The topic of critical complications of novel therapies for childhood cancer care and their management is addressed extensively in this Research Topic. This includes discussion of supportive care strategies for chimeric antigen receptor therapies (CART), such as simulation team-based training (Harden et al.) to improve management of toxicities, early diagnosis of neurologic complications of CART (ICANS) (Brown et al.), and cytokine release syndrome (CRS) (Baumeister et al.). Novel therapies in this field also include an expansion of supportive care interventions for critically ill children with cancer and post HCT, including CRRT (Elbahlawan et al.), non-invasive positive pressure ventilation (NIPPV) (Rowan et al.), bronchoscopy (Ahmad et al.), and ECMO (Ghafoor et al). Finally, the field has also implemented novel quality improvement interventions to improve outcomes, such as screening to identify delirium (Traube et al.), early mobility (Ghafoor et al.), and PEWS (Garza et al.). Over the past three years, the COVID-19 pandemic has presented additional challenges to caring for children with cancer and post-HCT (Ragoonanan et al.); this Research Topic similarly provides insight into how this new infection impacted this unique patient population.

### Early recognition of critical illness

Early recognition of critical illness leading to prompt interventions are likely contributing to improvements in mortality seen in critically ill pediatric oncology and post-HCT patients. Agulnik et al demonstrated that patients with longer duration of higher PEWS scores on the ward prior to PICU transfer have worse outcomes including higher mortality and fewer PICU-free days, ventilator-free days, and vasopressor-free days. The same group has also shown that implementation of PEWS empowered clinicians to speak up when concerned about their patient’s clinical status and improved overall (Graetz et al.) perceived quality of care during deterioration (Garza et al.).

Others in this collection similarly discuss the importance of early recognition of critical illness and prompt intervention. In a recent multi-center prospective point prevalence study, Traube et al. discovered that 45% of HCT patients experience delirium at some point in their transplant course. Delirium has been associated with worse outcomes in ICU patients (18). However, it’s impact on pediatric HCT patients in unknown and warrants further study. In another study, Brown et al. found that worsening Cornell Assessment of Pediatric Delirium (CAPD) scores were seen 24 to 72 hours prior to patients developing immune effector cell-associated neurotoxicity (ICANS) after chimeric antigen receptor (CAR) T-cell therapy. They stressed the importance of prompt recognition and intervention to prevent rapid neurologic deterioration. In a multi-center retrospective study of HCT patients treated with non-invasive ventilation (NIV), Rowan et al. found that 63% of patients failed NIV, requiring invasive mechanical ventilation (IMV). The most concerning finding was that patients who failed NIV had a very high rate of cardiac arrest when transitioning to IMV with 11% suffering cardiac arrest during intubation and another 3% suffering cardiac arrest just prior to intubation. This rate is much higher than the 1.7% seen in the general pediatric population (19). The authors found that patients who continued to have a respiratory rate greater than 40 after 4 hours of NIV were likely to fail NIV and require intubation. They hypothesized that earlier recognition of NIV failure may lead to earlier intubation and mitigate the risk of cardiac arrest during the procedure. The timing of intubation in these patients continues to be an important question that requires further study.

### Infections in POCC

Infections in HCT and oncology patients continue to be major challenges in this immunosuppressed population. Multiple case reports (Navazo et al.) demonstrated disseminated toxoplasmosis to be deadly in this vulnerable population (Lindell et al.). A case series of stenotrophomonas infection in pediatric oncology and HCT patients also demonstrated high mortality and fatal pulmonary hemorrhages (Zollner et al.). A case series of children with Dengue fever demonstrated how this infection can lead to life threatening hemophagocytic lymphohistiocytosis (HLH) (Singh et al.). Ferdjallah et al. reviewed the changing risk factors for infectious complications in HCT patients throughout their first year of transplant. This is helpful when evaluating HCT patients with possible infectious complications. Zinter et al. provides us with a road map for management of presumed sepsis in the HCT population. Zinter and Hume discuss the unique pulmonary immune response to infection in the HCT population, highlighting the complex factors that place this population at increased risk for pulmonary complications. These publications remind us of the seriousness of infectious complications in this immunosuppressed population. In addition to infections, non-infectious complications, often related to immune dysregulation, play an important role in the development of critical illness in HCT and CAR-T patients. Baumeister et al. provides us with an excellent review of complications related to immune dysregulation during HCT and CAR T-cell therapy.

### Respiratory failure and management

Pulmonary complications affect 25% of pediatric HCT patients, with nearly 10% requiring mechanical ventilation and represent a major cause of transplant-related mortality. Fitch et al. provides a thorough review of both infectious and non-infectious pulmonary complications post-transplant. The authors point out the importance of timing of complication in relation to the transplant in determining the differential diagnosis and cause. They also highlight significant knowledge gaps, particularly in non-infectious complications making them difficult to accurately diagnose and limitations in available treatments. Fan et al. additionally provide a review of one pulmonary complication, diffuse alveolar hemorrhage (DAH). Traditionally, DAH has had a very high mortality rate in HCT patients. This review discusses what is known about the pathophysiology of DAH as well as novel inhaled therapies to control bleeding.

Diagnosis of pulmonary complications in critically ill pediatric oncology and HCT patients can be complicated. While CT scans may be useful, they are often not specific, and travelling outside of the ICU when patients are unstable can be difficult. Debate continues over when and if patients should undergo invasive procedures such as bronchoscopy, bronchoalveolar lavage (BAL), and lung biopsy. Elbahlawan et al use a case report to illustrate the utility of lung biopsy in select patients. Ahmed et al show us through a retrospective chart review study that BAL is feasible and safe in these patients. In their center, BAL yielded a diagnosis in over 60% of patients and resulted in a clinical management change in over 69% of patients. To positively impact HCT outcomes, there is a need for improved understanding of the pathophysiology of pulmonary complications.

Supportive management of patients with respiratory failure is also an important topic in this field. Sallee et al. performed a secondary analysis of a retrospective multi-center study of HCT patients requiring mechanical ventilation for respiratory failure. They found that fluid overload was associated with increased risk of mortality. In patients treated with CRRT, the mortality risk was mitigated suggesting that intervention with CRRT may be helpful in improving survival rather than fluid overload simply being a marker of worse illness. These findings lay the groundwork for future studies addressing the optimal management of fluid overload in HCT patients.

Despite our limitations in understanding of pulmonary complications, there is a trend toward advocating for more aggressive management of respiratory failure in HCT and oncology patients. Ghafoor et al. describe an early mobilization program in their PICU. The authors were able to show that mobilizing intubated pediatric oncology and transplant patients was safe and feasible. While there are theoretical benefits to early mobilization of intubated patients, the true impact on outcomes needs further study.

### Multinational, multidisciplinary collaboration

The most encouraging finding in this collection of publications is the interest in developing a better understanding of the pathophysiology of critical illness in this population and provide critical care resources to support these vulnerable patients. The medical community seems to fortunately be breaking away from the self-fulfilling prophecy that these children have little chance of surviving critical illness and should not be offered critical care interventions. Publications in this Research Topic demonstrate multidisciplinary collaborations using multiple research methodologies to proactively address critical complications can significantly improve care and outcomes for these high-risk patients.

In addition to increasing publications in this Research Topic, multi-disciplinary, multi-center research collaboratives have been formed around the world to address these topics. The Pediatric Acute Lung Injury and Sepsis Investigator’s (PALISI) Network formed a Hematopoietic Cell Transplant – Cancer Immunotherapy subgroup to advance research in the field of onco-critical care in 2006 (20). More recently, a similar group was formed in Europe, PICU Oncology Kids in Europe Research Group (POKER) (21), and the St. Jude Global Critical Care Program (22) supports critical care for children with cancer in resource-limited settings worldwide. These groups work collaboratively with oncologists, transplant physicians, multidisciplinary professionals, and other relevant pediatric subspecialists. The presence of these groups is evidence for interest in the field and the importance of collaborative research to address its most difficult challenges.

## The future of our field

This Research Topic represents a new field in pediatric critical care—pediatric onco-critical care (POCC)—focused on improving care and outcomes for critically ill children with cancer and blood disorders. Over the past few years, multiple collaborative groups have developed POCC educational curricula (23), consensus research priorities (24), and quality and capacity indicators (25, 26). This Research Topic describing 30 articles on the critical complications in pediatric oncology and HCT marks the future of this growing field. This represents the beginning of the era of POCC—a global focus on major challenges in the management of critically ill children with cancer and blood disorders. Success in this new era will require a move from epidemiology to novel intervention leveraging global, multidisciplinary collaboration and specific attention to health equity.

The future of POCC must address ongoing clinical challenges described in this Research Topic, including early identification of complications, management of respiratory failure post-HCT, endothelial dysfunction, and multisystem organ failure. Future work must move from describing the epidemiology of these challenges to developing and testing novel interventions that improve outcomes.

This work must leverage a proactive, data-driven model to identify high-risk patient populations and discover new targeted therapeutic approaches. Such research in POCC must test, among others, bedside strategies to identify organ dysfunction syndromes, ventilatory strategies to improve survival in respiratory failure, and novel therapeutics to control inflammation and improve endothelial dysfunction. As PICU mortality for POCC patients declines, the impact of such interventions must be assessed beyond mortality, focusing on resource utilization, functional outcomes, and cost of care. As emphasized by work in this Research Topic, we must also look beyond the individual patient and evaluate how our care processes impact patient families, the care team, and health systems.

Overcoming these tough challenges in POCC will require multidisciplinary approaches, both in clinical management and research. Future work must emphasize interdisciplinary (oncology, ICU, etc) and interprofessional (nurses, physician, respiratory therapists, basic scientists, etc) collaborations to develop effective interventions and implement them as part of routine care. To succeed, clinicians and researchers must break out of silos to form productive, effective partnerships across disciplines and promote cross-fertilization of ideas and strategies. It is only through such diverse future collaborations that we can address the current challenges in POCCS.

Similarly, testing interventions to improve outcomes in POCC will require collaborations between institutions and countries. Children with cancer and critical illness, particularly with specific complications, are relatively rare; adequate power for any interventional trial will require global, institutional collaboration and joint research ventures. Networks such as POKER (21), the PALISI HCT Subgroup (20), and the St. Jude Global Critical Care Program (22) must be leveraged in large-scale collaborative research trials.

Although multiple challenges remain for POCC in high-resource settings (27), most children with cancer live in low-and middle income countries (28, 29), where critical illness is more common and results in worse outcomes (30). The future of POCC must intentionally address health equity in this field through research that specifically focuses on improving care and outcomes for critically ill children with cancer and blood disorders in resource-limited settings. This includes addressing therapeutic, resource, and implementation challenges that may be unique to POCC in these settings (26, 31, 32). This targeted focus is urgently needed to reduce global disparities in childhood cancer outcomes.

Finally, success in POCC must move beyond simply developing novel interventions that improve care processes and outcomes. These interventions must be usable beyond the research setting and be designed for implementation in real-world clinical environments. To accomplish this task, POCC researchers must collaborate with experts in the fields of implementation science, quality improvement, and health economics to design strategies that promote uptake and utilization of effective strategies in routine patient care. Similarly, discoveries in this field must be actively disseminated, both through open-access Research Topics such as this journal collection, as well as at academic conferences, through social media networks, and in clinician education. For lasting impact, discovery is insufficient; innovations must be actively incorporated into clinical practice on a global scale.

As curative therapies and treatment for childhood cancer and blood disorders improve over time, survival and quality of life depends on effective supportive care strategies to reduce complications of life-threating toxicities of therapy. The field of POCC addresses this need by combining multidisciplinary expertise across clinical, research, and methodologic fields to improve outcomes for critically ill children with cancer and blood disorders. This Research Topic describes the breadth and depth of this new field and lays the foundation for future work. We hope you will join us in the POCCS new frontier.

## Author contributions

AA, JM, and MS contributed to writing of the manuscript; all authors critically reviewed the manuscript and approved the final submission.
